# Effects of Liposomes Charge on Extending Sciatic Nerve Blockade of N-ethyl Bromide of Lidocaine in Rats

**DOI:** 10.1038/srep38582

**Published:** 2016-12-07

**Authors:** Qinqin Yin, Bowen Ke, Xiaobing Chen, Yikai Guan, Ping Feng, Guo Chen, Yi Kang, Wensheng Zhang, Yu Nie

**Affiliations:** 1 Laboratory of Anaesthesia and Critical Care Medicine, Translational Neuroscience Centre of West China Hospital, Sichuan University, Chengdu, Sichuan, 610041, P.R. China; 2 National Engineering Research Centre for Biomaterials, Sichuan University, Chengdu 610064, Sichuan, P.R. China; 3 Institution of Clinical Trials, West China Hospital, Sichuan University, Chengdu 610041, Sichuan, P.R. China; 4 Department of Anaesthesiology, West China Hospital, Sichuan University, Chengdu 610041, Sichuan, P.R. China

## Abstract

N-methyl bromide of lidocaine (QX-314) is a potential local anaesthetic with compromised penetration through cell membranes due to its obligated positive charge. Liposomes have been widely used for drug delivery with promising efficacy and safety. Therefore we investigated the local anaesthetic effects and tissue reactions of QX-314 in combination with anionic, cationic or neutral liposomes in rat sciatic nerve block model, and explored the effects of these liposomes on cellular entry of QX-314 in human embryonic kidney 293 cells. The results demonstrated that anionic liposomes substantially prolonged the duration of sensory (25.7 ± 8.3 h) and motor (41.4 ± 6.1 h) blocks of QX-314, while cationic and neutral ones had little effects. Tissue reactions from QX-314 with anionic liposomes were similar to those with commonly used local anaesthetic bupivacaine. Consistent with *in vivo* results, the anionic liposomes produced the greatest promotion of cellular entry of QX-314 in a time-dependent manner. In conclusion, ultra-long lasting nerve blocks were achieved by a mixture of QX-314 and anionic liposomes with a satisfactory safety profile, indicating a potential approach to improve postoperative pain management. The liposome-induced enhancement in cellular uptake of QX-314 may underlie the *in vivo* effects.

Local anaesthetics (LA) are widely used to alleviate pain caused by invasive procedures. Long-lasting LA will meet the great needs of post-operative pain management. However, traditional LA have short period of actions, usually less than 8 h^1^. Recently, N-methyl bromide of lidocaine (2-[(2,6-dimethylphenyl)amino]-N,N,N-triethyl-2-oxoethanaminium, also as known as QX-314) has emerged as a potential LA^2^,^3^. The analgesia produced by QX-314 in rodents is several fold longer than that by lidocaine, a traditional LA. However, the effect of QX-314 is limited by its poor ability of penetrating through lipid tissues. QX-314 is highly hydrophilic because of its obligated positive charge. It is difficult for QX-314 to diffuse from the injection sites (tissues in the vicinity of nerve) to reach the action sites (the sodium channels on the inner surface of neural membranes) through lipid barriers such as muscles, epineruim, myelin sheath, and cell membranes. Effective anaesthesia requires QX-314 at relatively high concentrations, which are associated with local tissue toxicities^4^.

Recently, Sagie and Kohane used surfactants, such as those are used in transdermal drug delivery, to enhance the diffusion of QX-314 across the lipid barriers. QX-314 with anionic sodium octyl sulfate provided sensory blocks for approximately 15 h; while co-injection of QX-314 with cationic octyltrimethylammonium bromide or neutral polyoxyethy-lene (20) sorbaitan monolaurate produced sciatic nerve blocks only up to 7 h and 5 h, respectively^5^. The pattern of prolongation was demonstrated as anionic ≫ cationic > neutral surfactants; the anionic surfactant was proved the most potent. The underlying mechanisms remain unknown, but are postulated to be associated with the interaction among charges, hydrophobicities, and other physiochemical properties of QX-314 and surfactants. It is known that most surfactants could cause local tissue toxicities, including inflammation and neuron necrosis, which can lead to permanent nerve dysfunction, so safety would be a major concern for peri-neural administration^6^.

Liposome has been employed for the delivery of drugs including small molecular drugs, proteins and genes^7^,^8^,^9^,^10^, showing promising efficiency and bio-safety on account of its bio-membrane mimetic structure and component. In clinical settings, the only commercial-available sustained release formulation of local anesthetics to date is multivesicular liposomal bupivacaine (Exparel^®^), which provided long-lasting analgesia in wound infiltration^11^. Compared with surfactants, liposomes have advantages in terms of tissue compatibility and local tissue reaction that have been confirmed in clinical practices^12^.

In our previous reports, anionic and cationic PEGylated liposomes were synthesized with charges from hemisuccinated and lysine in cholesterol derivatives, demonstrating prolonged retention release profile, low cytotoxicity, and improved cellular uptake^13^. Deduced from the above studies, QX-314 may electrostatically interact with liposomes that carry the same or opposite charges, and attach to liposomes which would help enter nerve fibers, facilitating prolonged anaesthesia effects. We hypothesized that our designed liposomes would prolong the nerve blocks from QX-314 *in vivo*, and enhance cellular entry of QX-314 *in vitro*.

## Results and Discussion

### Characterization of different charged liposomes

**Figure 1:**
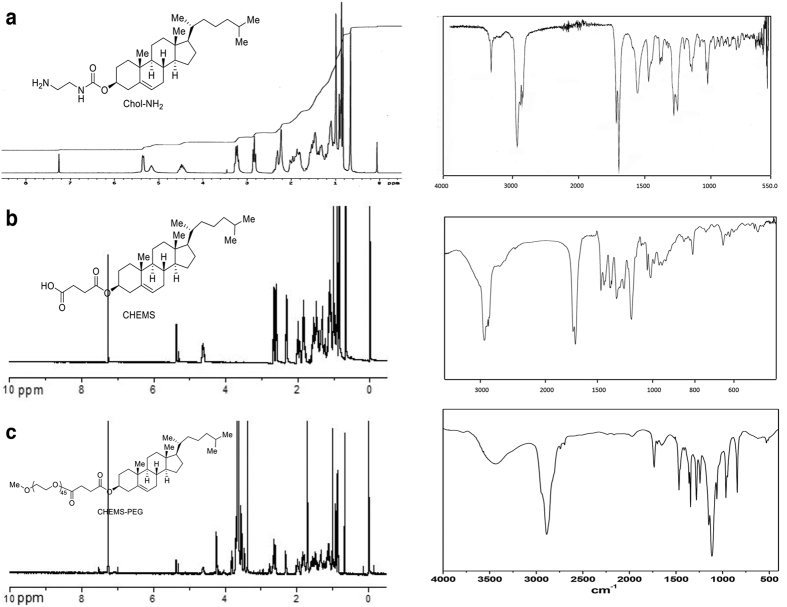
Structure confirmation of cationic, anionic and neutral PEGylated cholesterol derivatives by ^1^H-NMR (left) and IR (right). (**a**) N1-cholesteryloxycarbonyl-1, 2-diaminoethane (Chol-NH_2_), (**b**) cholesterol hemisuccinate (CHEMS) and (**c**) PEGylated cholesterol derivative (CHEMS-PEG).

**Figure 2:**
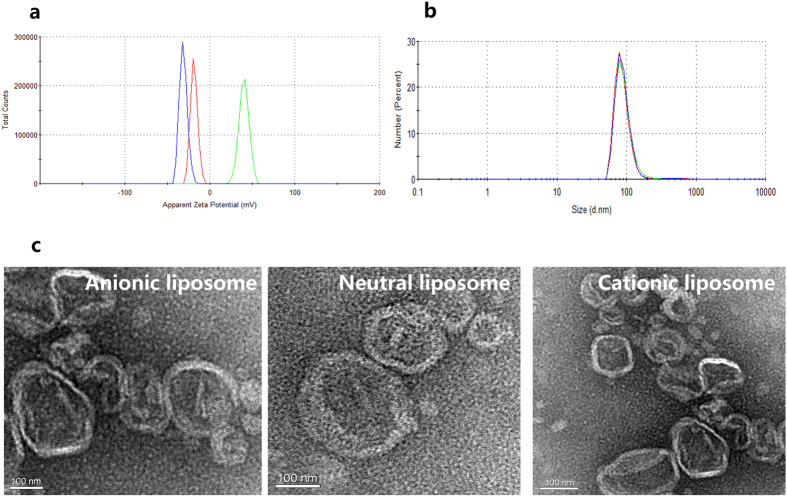
Characterization of anionic, neutral and cationic liposomes. (**a**) Zeta potential (AL: blue, NL: red, CL: green) and (**b**) size distribution (AL: blue, NL: red, CL: green) of various charged liposomes. (**c**) Morphology observation by transmission electron microscopy. AL: anionic liposomes, NL: neutral liposomes, CL: cationic liposomes.

**Figure 3:**
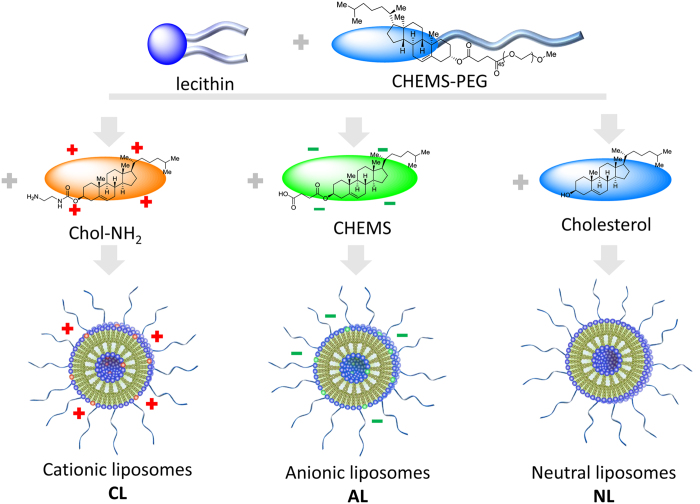
Anionic, cationic and neutral liposomes formulation.

Different charged PEGylated liposomes were characterized under the same conditions. The results of size distribution and zeta potential are presented in Table 1 and Fig. 1. It was obvious that three kinds of liposomes showed similar diameter around 240~270 nm (Table 1), and the morphology observed from microscopy (Fig. 2c) was corresponded to that obtained from dynamic light scattering (DLS) determination, with spherical shape. No size and morphology difference was detected between liposomes composed of different components. Zeta potential of liposomes containing N_1_-cholesteryloxycarbonyl-1, 2-diaminoethane (Chol-NH_2_) and anionic cholesterol hemisuccinate (CHEMS) were +39 mV and −31 mV, respectively, while liposomes composed of PEGylated cholesterol derivative (Chol-PEG) was much lower (−9 mV). Thus these three kinds of liposome were defined as cationic liposomes (CL), anionic liposomes (AL) and neutral ones (NL) (Fig. 3).

### Prolonged nerve blockade in QX-314 mixed with anionic liposomes

**Figure 4:**
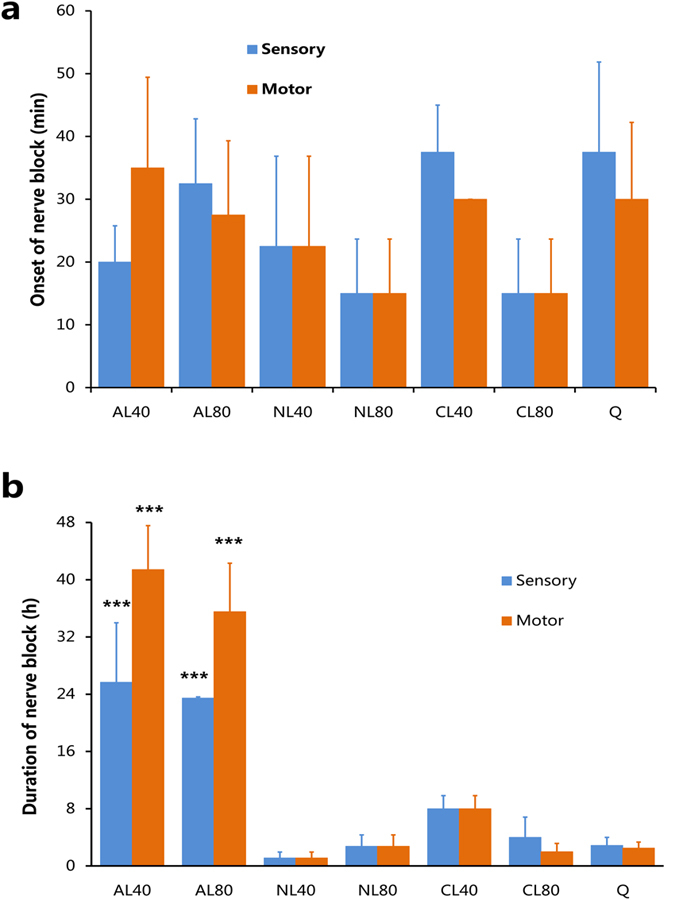
The onset times and durations of sciatic nerve blocks in rats (sample size of eight). (**a**) The onset times and (**b**) the durations. ***statistically longer (*P* < 0.001) than the groups of NL40 (25 mmol/L QX-314 in 40 mmol/L neutral liposomes), NL80 (25 mmol/L QX-314 in 80 mmol/L neutral liposomes), CL40 (25 mmol/L QX-314 in 40 mmol/L cationic liposomes), CL80 (25 mmol/L QX-314 in 80 mmol/L cationic liposomes), and Q (25 mmol/L QX-314 alone). Data was presented as *mean* ± *SEM.*

To investigate the effects of liposomes on enhancing the nerve blockades from QX-314, 25 mmol/L QX-314 was peri-sciatically injected alone (Q) or with 40 mmol/L anionic liposomes (AL40), 80 mmol/L anionic liposomes (AL80), 40 mmol/L neutral liposomes (NL40), 80 mmol/L neutral liposomes (NL80), 40 mmol/L cationic liposomes (CL40), or 80 mmol/L liposomes (CL80). The negative control, Saline, did not elicit any detectable analgesia or muscle strength deficits. Peri-sciatic injection of 0.5% bupivacaine, the positive control, produced effective sensory and motor blockade that lasted for 4.0 ± 0.8 h and 3.6 ± 1.3 h, respectively, consistent with literature^14^. 25 mmol/L QX-314 resulted in slow-onset, short-lasting nerve blockades (sensory blockade: 2.9 ± 1.1 h, motor blockade: 2.5 ± 0.8 h; Fig. 4) similar to the observations from a previous research^4^. Co-application of cationic, anionic or neutral liposomes did not significantly accelerate the onset of sensory and motor block. (Fig. 4a, *P* = 0.502 among groups). Compared with cationic and neutral liposomes, anionic ones (AL40) provided significantly longer sensory (25.7 ± 8.3 h, *P* < 0.001) and motor (41.4 ± 6.1 h, *P* < 0.001) nerve blockade; increasing the concentration of liposomes to 80 mmol/L did not lead to further prolongation of duration (AL40 *vs.* AL80, *P* = 0.997; NL40 *vs*. NL80, *P* = 0.664; CL40 *vs.* CL80, *P* = 0.39) (Fig. 4b). No statistical difference was detected in duration of nerve blocks among Q, CL40, CL80, NL40, and NL80 groups (Fig. 4b). These *in vivo* data suggested that anionic liposomes substantially prolonged the duration of QX-314, whereas cationic and neutral liposomes had little effect on extending the effectiveness of QX-314.

Among cationic, anionic, and neutral liposomes, anionic liposomes was the most potent to enhance the effects of nerve blockades from QX-314. Similar to this pattern of prolongation, anionic surfactants prolonged the duration of sensory nerve block of QX-314 to a greater extent than cationic and neutral surfactants^5^. The prolonged nerve blocks could be attributed to the interaction between QX-314 and anionic additives. There are studies that demonstrate electrostatic interaction between LA and anionic liposomes or lipid tissues. Compared with the commercial neutral lipid emulsion (Intralipid), greater interaction between negatively charged liposomes dispersion and commonly used LA (bupivacaine, prilocaine, and lidocaine) was discovered in an electrokinetic chromatographic study^15^. Bupivacaine interacts with negatively charged cardiolipin head groups through electrostatic effects^16^. We know for a fact that bupivacaine exists in forms of both free bases and cations under physiological conditions. To produce anesthetic effects, it must penetrate through the lipid bilayer cellular membranes in the form of free bases, before binding to sodium channels from the cytoplasm side in the form of cations^17^. The molecular structure difference between traditional LA and QX-314 is that the latter is positive charged under physiological conditions. Therefore QX-314 has difficulty in passive penetration through cellular membranes, as reflected by the slow onset (Fig. 4a). However, QX-314 would be readily attached to the surface of anionic liposomes by electrostatic force for carrying a positive charge.

It is well known that liposomes have good affinity to cell membranes and lipid tissues^7^,^18^,^19^. Previously, hydrocortisone or insulin administered with liposomes resulted in targeted delivery through skins^20^. Charged liposomes has been used in transdermal delivery of drugs^21^. Upon peri-neural injection, anionic liposomes would penetrate through lipid barriers such as muscles, epineurium, myelin, and cell membranes while carrying QX-314, helping QX-314 reach the intracellular action sites.

In our study, the durations of sensory blockade did not exceed that of motor blockades no matter what kind of liposomes was co-applied (Fig. 4b). However, the durations of sensory nerve blockade was obviously longer than the motor blockade when QX-314 was injected with anionic, cationic, or neutral surfactants. Therefore the sensory-selectivity of nerve block was probably due to chemical effects or other reasons, rather than electrostatic effects.

**Figure 5:**
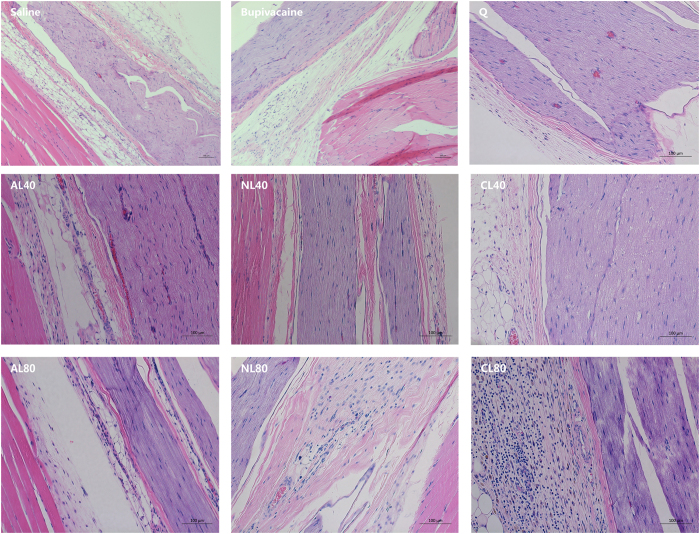
The histomorphological changes of sciatic nerves and the adjacent tissues two weeks after peri-neural injection. Bupivacaine: 0.5% bupivacaine hydrochloride; Q: 25 mmol/L QX-314 alone; AL40 (25 mmol/L QX-314 in 40 mmol/L anionic liposomes); AL80 (25 mmol/L QX-314 in 80 mmol/L anionic liposomes); NL40 (25 mmol/L QX-314 in 40 mmol/L neutral liposomes), NL80 (25 mmol/L QX-314 in 80 mmol/L neutral liposomes), CL40 (25 mmol/L QX-314 in 40 mmol/L cationic liposomes), CL80 (25 mmol/L QX-314 in 80 mmol/L cationic liposomes).

No signs of systemic toxicity were observed. Sensory or motor function deficit was absent in the un-treated limbs, indicating insignificant systemic distribution or low systemic toxicity^22^. The gross appearance of sciatic nerves and the adjacent tissues were normal. The histological examinations revealed moderate to severe inflammation cell infiltration in group CL80 and NL80; but tissue reaction to the rest test solutions was similar with that to 0.5% bupivacaine (Fig. 5). These morphological results indicated that the tissue irritations from 25 mmol/L QX-314 mixing with 40 mmol/L liposomes were acceptable.

### Improved cellular accumulation of QX-314 by liposomes

**Figure 6:**
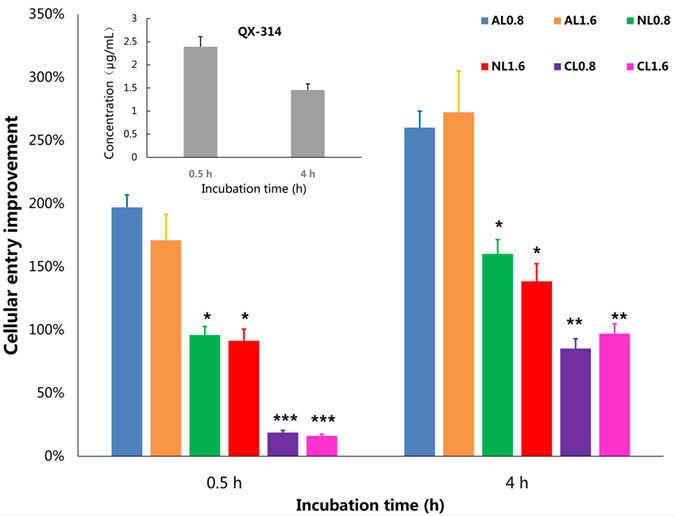
Liposomes-induced improvement of cellular uptake of QX-314 in HEK293 cells. The intracellular concentration of QX-314 after 0.5 h and 4 h of incubation with 0.5 mmol/L QX-314 alone were statistically similar (in the upper left region of this figure). Anionic liposomes markedly increase the intracellular concentration of QX-314; co-incubation of neutral liposomes have limited effects on improving cellular uptake of QX-314; cationic liposomes, however, decrease cellular entry of QX-314 after 0.5 h of co-incubation. AL0.8: 0.8 mmol/L anionic liposomes; AL1.6: 1.6 mmol/L anionic liposomes; NL0.8: 0.8 mmol/L neutral liposomes; NL1.6: 1.6 mmol/L neutral liposomes; CL0.8: 0.8 mmol/L cationic liposomes; CL1.6: 1.6 mmol/L cationic liposomes. *P < 0.05; **P < 0.01; ***P < 0.001 compared to anionic liposomes at the corresponding concentration, respectively.

The animal experiments demonstrated that anionic liposomes substantially prolonged the sciatic nerve blocks of QX-314. In order to illustrate whether liposomes improved cellular entry of QX-314, HEK293 cells were incubated with QX-314 alone or with anionic, neutral or cationic liposomes. When QX-314 was used alone, increasing the incubation time from 0.5 h to 4 h did not result in elevation of the intracellular QX-314 concentration (*P* = 0.818), consistent with the slow onset *in vivo*. In the presence of liposomes, however, the cellular uptake of QX-314 was obviously (but not statistically) improved as the incubation time prolonged. After co-incubation of 0.5 h, anionic liposomes substantially increased the intracellular concentration of QX-314; neutral liposomes have little effect on improving cellular uptake of QX-314; whereas cationic liposomes reduced the intracellular concentration of QX-314. The trend of the three kinds of liposomes on improving cellular entry of QX-314 remained the same when the concentrations of liposomes were doubled or the incubation time was prolonged to 4 h (Fig. 6). In summary, the anionic liposomes produced the greatest, time-dependent promotion of cellular entry of QX-314.

We noticed that increasing the concentration of liposomes from 40 mmol/L to 80 mmol/L did not result in greater prolongation of nerve blockades, nor further increase in cellular uptake of QX-314. We are not sure about the reasons. One possibility could be the lack of obvious linear relationship between liposome concentrations and the effects on prolongation of nerve blockades. 80 mmol/L may be at (or after) the upper inflection point of the dose-effect curve. Another possibility might be the chemophysical characteristics of liposomes. Because we found in the results that this trend was the same for all three kinds of liposomes, indicating that the ineffectiveness of cationic and neutral liposomes was probably due to their intrinsic chemophysical properties.

Anionic liposomes enhanced the cellular uptake, and prolonged the nerve block effects of positive charged local anaesthetic QX-314 via simple mixing method. The multivesicular liposomal bupivacaine produced ultra-long lasting infiltration anaesthesia by sustained release of bupivacaine from a honey comb like structure of internal liposomal chambers^23^, however its effects in nerve blocks is less impressive in animals models^24^. Bupivacaine loaded liposomes produced rat sciatic nerve block for 4 h, compared with 2 h by bupivacaine hydrochloride. Using the same animal model, QX-314 mixed with anionic liposomes facilitated effective sensory blockade that lasted longer than 24 h. One explanation could be that the detouching of QX-314 from the surfaces of liposomes is easier and faster than the release of bupivacaine from internal liposomal chambers. Therefore the drug flux that enters the target sites (neurons) will be greater, and the drug flux is critical to produce the effects of nerve block^17^. The simply mixing method may have advantages over traditional encapsulation method. The preparation of QX-314+liposomes mixtures is easier than encapulating QX-314 into liposomes; and the initial dose of QX-314 will not be limited by the encapsulation rate.

## Conclusion

In conclusion, mixing of QX-314 with anionic liposomes produced ultra-long lasting sciatic nerve blocks in rats without systemic toxicity and local tissue injury, indicating a potential approach to improve postoperative pain management. The substantial increase in cellular accumulation of QX-314 by anionic liposomes may attribute to the prolongation effects *in vivo*.

## Materials and Methods

### Materials

Cholesterol (Chol), methoxypoly (ethylene glycol) (mPEG, MW = 2000 Da), cholesteryl chloroformate, 1, 2-diaminoethane and dicyclohexylcarbodiimide (DCC) were purchased from Sigma-Aldrich Chemical Co. (USA). Three cholesterol derivatives (Chol-NH_2_, CHEMS and Chol-PEG) were synthesized in our group. Soybean phosphatidylcholine (PC) for injection was obtained from Tywei Pharmaceutical Co. Ltd. The N-ethyl bromide of lidocaine (QX-314, C_16_H_27_BrN_2_O, molecular weight: 343 g/mol) was purchased from Sigma Aldrich, Shanghai, China. Human embryonic kidney 293 cells (HEK293) were purchased from Shanghai Institute of Biochemistry and Cell Biology (Chinese Academy of Sciences, Shanghai, China). Cell culture media (RPMI 1640), fetal bovineserum (FBS), trypsin, and antibiotics were purchased from Gibco BRL (USA). 0.75% Bupivacaine hydrochloride solutions were purchased from Jiang Su Heng Rui Medicine Co., Ltd. (Lianyungang, China). All the other chemicals of analytical grade were purchased from local commercial suppliers.

### Synthesis of different charged cholesterol derivatives

#### Synthesis of cationic cholesterol derivative: N_1_-cholesteryloxycarbonyl-1, 2-diaminoethane (Chol-NH_2_)

To a solution of cholesteryl chloroformate (4.0 g, 8.8 mmol) in 20 mL anhydrous chloroform was added 1, 2-diaminoethane (3.2 mL, 48 mmol) in 20 mL anhydrous chloroform dropwise at 0 °C. The mixture was stirred at room temperature for 4 h. Water was added to cool the resultant, which was extracted with dichloromethane. The Combined organic layers were washed by saturated sodium carbonate and saturated brine, and then dried with sodium sulfate. Organic solvents were removed by a rotary evaporator and the crude product was purified by flash chromatography on silica gel (DCM/MeOH 85:15, with trace TEA) yielding the product (2.9 g, 68% yield) as a light yellowish solid. R_f_ = 0.15 (DCM/MeOH/85:15); ^1^H NMR (400 MHz, CDCl_3_): δ = 0.67 (s, 3 H; H-18’), 0.86 (d, 6 H; H-26’, H-27’), 0.90 (d, 3 H; H-21’), 1.02 (s, 3 H; H-19’), 0.99-1.69 (m, 21 H; H-1’, H-4’, H-9’, H-11’, H-12’, H-14’, H-15’, H-16’, H-17’, H-20’, H-22’, H-23’, H-25’), 1.73-2.11 (m, 5 H; H-2’, H-7’, H-8’), 2.12-2.41 (m, 2 H; H-24’), 2.84 (t, 2 H; H-2), 3.26 (q, 2 H; H-1), 4.48-4.50 (m, 1 H; H-3’), 5.21 (bs, 1 H CholOCON*H*), 5.36 (d, 1 H); IR: 3338, 2944, 2867, 1715, 1696, 1551, 1468 cm^−1^; HRMS: calcd for C_30_H_52_N_2_O_2_ [m + H]^+^: 473.4107, found [m + H]^+^ 473.4120 (Fig. 1a).

#### Synthesis of anionic cholesterol hemisuccinate (CHEMS)

To a solution of cholesterol (3.9 g, 10 mmol) in anhydrous pyridine (40 mL) was added succinic anhydride (3.0 g, 30 mmol). The mixture was refluxed at 90 °C for 24 h, in the atmosphere of nitrogen. The residue was washed with a solution (HCl/distilled ice water 5:95, v/v). The product (4.6 g, 88% yield) was obtained by recrystallization from acetone. Melting point: 185 °C. IR γ/cm^−1^: 2946, 2867, 1731, 1710, 1467, 1438 cm^−1^; ^1^H NMR (CDCl_3_, δ ppm): 0.66 (s, 3 H, H-18), 0.90 (s, 3 H, H-21), 2.33 (d, 2 H, H-4), 2.60-2.62 (m, 2 H, -COCH_2_), 2.67-2.69 (m, 2 H, -CH_2_CO), 5.36 (t, 1 H, H-6) (Fig. 1b).

#### Synthesis of neutral PEGylated cholesterol derivative (Chol-PEG)

To a solution of CHEMS (3.6 g, 7.5 mmol) in 30 mL anhydrous CH_2_Cl_2_ was added sulfonyl chloride (1 g, 7.5 mmol) under nitrogen stream. The mixture was refluxed for 5 h. mPEG2000 (3.0 g, 1.5 mmol) was added into the solution and the reaction continued for another 5 h. The mixture was condensed and precipitated in diethyl etherthrice and the precipitate was recrystallized in anhydrous ethanol thrice. The crude product was purified by dialysis to remove excessive mPEG (10 kDa cut-off) to give the pure compound (3.3 g, 88% yield). IR: 2890, 1734, 1460 cm^−1^; ^1^HNMR (CDCl_3_, δ ppm): 0.67 (s, 3 H, H-18), 0.90 (s, 3 H, H-21), 2.34 (d, 2 H, H-4), 2.60 (m, 2 H, -COCH_2_), 2.66-2.68 (m, 2 H, -CH_2_CO), 3.38 (s, 3 H, -CH_3_), 3.64 (m, 176 H, -OCH_2_O), 5.37 (t, 1 H, H-6) (Fig. 1c).

### Preparation of various charged liposomes and drug addition

Liposomes were prepared by traditional solvent injection method with different composition of PC, Chol, Chol-PEG and different charged components (Chol-NH_2_ and CHEMS), respectively (details about formulations were listed in Table 1). In brief, all components were dissolved in about 50 μL of ethanol, and then injected into 1 mL of phosphate buffer saline under fast stirring. These empty liposomes were formed as cationic liposomes (CL), anionic liposomes (AL) and neutral ones (NL) after the suspension was stirred at room temperature for half hour with lipid concentration as 80 mmol/L. Commonly, the liposome formulations were stored at 4 °C. Mixture of liposomes and QX-314 solution was prepared by gently dissolving drug powder in corresponding liposome solutions. The final mixture suspension was 25 mmol/L QX-314 in 40 mmol/L or 80 mmol/L cationic, anionic, or neutral liposomes, respectively. The pH values of obtained solutions were ranged from 6.5 to 7.9, measured by S40 Sevenmulti^TM^ pH meter (Mettler Toledo, USA). Bupivacaine hydrochloride solution was diluted with saline (Qing Shan Li Kang Pharmaceutical, Co., Ltd., Chengdu, China) to obtain 0.5% (15 mmol/L) bupivacaine as the positive control. Based on previous researches^4^,^5^ and our preliminary experiments, QX-314 was used at 25 mmol/L, and the co-injected liposomes were at 40 mmol/L or 80 mmol/L in animal experiments.

### Size distribution, morphology and zeta potential

The particle size distribution and surface charge of liposomes (0.5 mg/mL) in phosphate-buffered saline (PBS, pH 7.4) were measured by dynamic laser-light scattering (DLS) using Zetasizer (Nano ZS, Malvern Instruments Ltd., UK). Liposomes were placed on copper grid films and stained with 2% (w/v) phosphotungstic acid for morphological observation by transmission electron microscopy (TEM, JEM-100CX, JEOL, Japan).

### Animal experiments

The animal experiments were performed in accordance with the guide for the care and use of medical laboratory animals (Ministry of Health, China). All animal procedures were approved by the Institutional Animal Experimental Ethics Committee of Sichuan University (Chengdu, China, Approval file No. 2015014 A). Sprague-Dowley rats weighted 200~300 g were housed in a 12 h light/12 h dark cycle with free access to food and water. Animals were acclimated for one week before formal test, and randomized into nine groups with 8 rats each.

Baselines of sensory and motor function were measured for three consecutive days prior to experiments (see below). Rats with normal sensation and muscle strength received 0.2 mL of peri-sciatic nerve injection of the following test solutions: 25 mmol/L QX-314 (Q), 25 mmol/L QX-314 in 40 mmol/L anionic liposomes (AL40), 25 mmol/L QX-314 in 80 mmol/L anionic liposomes (AL80), 25 mmol/L QX-314 in 40 mmol/L neutral liposomes (NL40), 25 mmol/L QX-314 in 80 mmol/L neutral liposomes (NL80), 25 mmol/L QX-314 in 40 mmol/L cationic liposomes (CL40), or 25 mmol/L QX-314 in 80 mmol/L cationic liposomes (CL80). 0.5% bupivacaine hydrochloride and saline served as positive and negative control, respectively.

#### Sciatic nerve block

Sciatic nerve block was performed as previous described^5^,^25^. Inhaled with 1~2% isoflurane, rats were placed laterally, a 29-Gauge needle was introduced at the one-third distance of the imaginary line connecting the greater trochanter and ischial tuberosity (caudal to the greater trochanter). 0.2 mL of test solution was injected once the tip of the needle encountered the ischium. Observers were blinded to treatments animals received.

#### Evaluation of sensory and motor function

Response to noxious heat was used to evaluate the sensory blockade^5^, and the term “thermal nociceptive” is abbreviated to “sensory” for sake of brevity. The paw of the injected limb was gently placed on a 56 °C metal plate. The time from the placement of paw to the moment of paw withdrawal was the paw withdrawal latency (PWL), which represents the degree of sensory blockade. The baselines were approximately 2 s; the cut off time was set at 12 s in order to avoid tissue injury; and PWL exceeded 7 s was considered effective sensory nerve blockade.

Motor function was measured by the extensor postural thrust test^5^. A rat was vertically held so that the injected limb was placed on to an electronic balance. The value displayed by the balance was the weight borne that the limb could exert. Effective motor blockade was defined as the percentage of suppression of weight borne exceeding 50%, calculated as (baseline - test value)/baseline (%).

Sensory and motor blocks of both hind limbs were evaluated at 10 min, 30 min, 1 h after nerve block, and every 1 h thereafter within 4 h, then every 2 h within 12 h, and then every 12 h till complete recovery. The onset time of block was the first test point when effective sensory or motor block was manifested; the offset time was the first test point when effective nerve blockade disappeared; the duration of block was defined as the interval between the onset and the offset time.

#### Toxicity assessments

All rats were inspected during the injection and for the following two weeks for signs of systemic toxicity including restless, tremor, convulsion, seizure, or death; meanwhile they were also observed for behavioral evidence of local irritations such as muscle spasm, necrosis of injection site, self-mutilation, paw-licking or permanent paralysis^4^,^26^.

Two weeks after sciatic nerve block, animals were euthanized by intraperitoneal injection of pentobarbital at lethal doses. The sciatic nerve and adjacent tissues were harvest, stored in 10% formaldehyde solution, and then embedded in paraffin before been sliced into 5 μm slices. Hematoxylin–eosin staining was performed; histological evaluation was conducted using BX51 microscope system (Olympus, Tokyo, Japan). A 0-4 scale was used to semi-quantitatively evaluate the degree of inflammation, necrosis, degeneration and vacuolation within epineurium and in the adjacent muscles, where 0 = normal; 1 = 0~25% of area involved; 2 = 25%~50% of area involved; 3 = 50% to 75% involved; and 4 = 75% to 100% involved^27^.

### Cellular experiments

Human embryonic kidney 293 cells (HEK293) were used to investigate cellular entry of QX-314^14^. Test solutions were initially prepared as in animal experiments, then 1:50 diluted with PBS. The final concentration of QX-314 in cellular experiments was 0.5 mmol/L, and liposomes was 0.8 mmol/L or 1.6 mmol/L. Cells were mounted in six-well dishes, incubated with diluted test solutions for 30 min or 4 h, and then washed with 2 ml of PBS carefully. Then 1 mL 0.25% pancreatin was added to release cells from the plates. Suspension in each well was centrifuged at 1000 rpm for 3 min. The supernatant was replaced by 500 μL PBS. Cells were break down by repeated freezing (−180 °C) and thawing (37 °C) for five times. Suspension containing cell debris was centrifuged at 25000 rpm for 15 min to separate protein, 100 μL of the supernatant was mixed with 900 μL acetonitrile, and the concentration of QX-314 in the mixture was measured by liquid chromatograph-mass spectrometer (LC-MS).

The LC-MS system consisted of the following components: an Agilent 1260 High-performance liquid chromatography and Agilent 6460 mass spectrometer with an ESI source. 5 μL of the extracted sample was injected onto a C18 analytical column (3.0 × 100 mm, 3.5 mm particle size, ZORBAX Extend-C18, Agilent). The mobile phase consisted of solvent A 5 mmol/L ammonium acetate in water and solvent B 100% acetonitrile (70:30, v/v). The flow rate was 0.3 mL/min. The column temperature was maintained at 25 °C. MS conditions: Positive ions were monitored using multiple reactions monitoring (MRM). The following MS/MS ion transition was monitored ([M+H]^+^): QX314 m/z 263.0-86.1, retention time: 2.70 min; The following MRM parameters were found to give the best sensitivities: the source temperature was set to 300 °C, and the sheath gas temperature was 250 °C. The capillary voltage was 3500 V, The dwell time for each transition was 500 ms. The sheath gas flow was 11 L/min. The fragmentor voltage was 136 V and the collision energy was 24. The lower limits of quantification were 100 ng/mL for QX-314 with a range of reliable response from 1 to 100 ng/ml (r^2^ > 0.99).

To assess the liposome-induced improvement in cellular entry of QX-314, the concept of cellular entry improvement was introduced. It is the percentage of intracellular concentration of QX-314 in groups of QX-314 + liposomes to that in group of QX-314 alone.

### Statistical analysis

Statistical analysis was conducted with SPSS (version 16.0, SPSS Inc.) Normal distribution of data was tested before comparisons. Nerve block durations, intracellular concentration, and the cellular entry improvement among groups were compared by a one-way ANOVA followed by *Bonferroni post hoc* test in order to reduce the type I error rate. *Kruskal-Wallis* test was conducted for inflammation score comparison among groups. Independent-sample *T* test was employed to analyze the difference of cellular uptake improvement between two groups of liposomes. Statistical tests were two-tailed, difference was considered significant when *P* value below 0.05. Characterizations of liposomes and histological photographies were obtained following the earlier description in this section. Scheme of liposome formulations were originally drawn with Powerpoint (Microsoft for windows, USA). The rest figures (animals and cellular experiments results) were firstly generated in Excel (Microsoft for windows, USA). All figures were combined, format-converted using Photoshop CS5 (Adobe, USA).

## Additional Information

**How to cite this article:** Yin, Q. *et al*. Effects of Liposomes Charge on Extending Sciatic Nerve Blockade of N-ethyl Bromide of Lidocaine in Rats. *Sci. Rep.* **6**, 38582; doi: 10.1038/srep38582 (2016).

**Publisher's note:** Springer Nature remains neutral with regard to jurisdictional claims in published maps and institutional affiliations.

## Figures and Tables

**Table 1:** Component, size distribution and zeta potential of charged liposomes.

Type of Liposome	Molecular radio	Particles size (nm)	Zeta potential (mV)
PC/Chol/CHEMS-PEG (neutral liposome, NL)	40/17/3	249.3 ± 12.3	−9.6 ± 0.4
PC/Chol/CHEMS/CHEMS-PEG (anionic liposome, AL)	40/8.5/8.5/3	275.3 ± 10.6	−31.2 ± 0.9
PC/Chol/Chol-NH_2_/CHEMS-PEG (cationic liposome, CL)	40/8.5/8.5/3	271.6 ± 13.8	39.6 ± 1.2
